# Personalized matched targeted therapy in advanced pancreatic cancer: a pilot cohort analysis

**DOI:** 10.1038/s41525-022-00346-5

**Published:** 2023-01-20

**Authors:** Justin Shaya, Shumei Kato, Jacob J. Adashek, Hitendra Patel, Paul T. Fanta, Gregory P. Botta, Jason K. Sicklick, Razelle Kurzrock

**Affiliations:** 1 Division of Hematology and Oncology, Department of Medicine, University of California San Diego Moores Cancer Center, La Jolla, CA USA; 2 Center for Personalized Cancer Therapy, University of California San Diego Moores Cancer Center, La Jolla, CA USA; 3 Department of Oncology, The Sidney Kimmel Comprehensive Cancer Center at The Johns Hopkins Hospital, Baltimore, MD USA; 4 Department of Surgery, Division of Surgical Oncology, University of California San Diego, UC San Diego Health, San Diego, CA USA; 5 Department of Pharmacology, University of California San Diego, UC San Diego Health, San Diego, CA USA; 6 Genomic Sciences and Precision Medicine Center, Medical College of Wisconsin, Milwaukee, WI USA; 7 WIN Consortium, Paris, France; 8 University of Nebraska, Lincoln, NE USA

**Keywords:** Predictive markers, Tumour biomarkers

## Abstract

Despite progress, 2-year pancreatic cancer survival remains dismal. We evaluated a biomarker-driven, combination/N-of-one strategy in 18 patients (advanced/metastatic pancreatic cancer) (from Molecular Tumor Board). Targeted agents administered/patient = 2.5 (median) (range, 1–4); first-line therapy (*N* = 5); second line, (*N* = 13). Comparing patients (high versus low degrees of matching) (matching score ≥50% versus <50%; reflecting number of alterations matched to targeted agents divided by number of pathogenic alterations), survival was significantly longer (hazard ratio [HR] 0.24 (95% confidence interval [CI], 0.078–0.76, *P* = 0.016); clinical benefit rates (CBR) (stable disease ≥6 months/partial/complete response) trended higher (45.5 vs 0.0%, *P* = 0.10); progression-free survival, HR, 95% CI, 0.36 (0.12–1.10) (*p* = 0.075). First versus ≥2nd-line therapy had higher CBRs (80.0 vs 7.7%, *P* = 0.008). No grade 3–4 toxicities occurred. The longest responder achieved partial remission (17.5 months) by co-targeting MEK and CDK4/6 alterations (chemotherapy-free). Therefore, genomically matched targeted agent combinations were active in these advanced pancreatic cancers. Larger prospective trials are warranted.

## Introduction

Despite advances in the management of advanced pancreatic cancer, outcomes remain dismal. With current systemic therapy options, 2-year overall survival (OS) is less than 10% in patients with metastatic disease, with substantial toxicity from systemic chemotherapy^1,2^. Frontline cytotoxic chemotherapy options rely on FOLFIRINOX (5-fluorouracil, leucovorin, irinotecan, oxaliplatin) and gemcitabine/nab-paclitaxel backbones^2^. There is a clear paucity of targeted therapy options.

The most common genomic alterations noted in metastatic pancreatic cancer specimens are in *KRAS*, *TP53*, *CDKN2A*, and *SMAD4* genes. The products of these genes are believed to be difficult to inhibit with targeted therapies^3^. Several phase II/III trials of targeting agents (bevacizumab^4^, cetuximab^5^, trametinib^6^, selumetinib^7^, and tipifarnib^8^) failed to show efficacy. Importantly, however, these trials accepted all-comers and patients were not specifically selected for targeted treatment by their tumor’s genomic anomalies. While the combination of gemcitabine and the targeted epidermal growth factor receptor (EGFR) inhibitor erlotinib showed a statistically significant improvement in medial overall survival (OS) compared to gemcitabine alone^9^, the improvement in median OS was less than two weeks and patients had considerable skin toxicity. On the other hand, the PARP inhibitor olaparib has shown activity in pancreatic cancers harboring germline *BRCA* mutations^10^. Taken together, most previous pancreatic cancer targeted therapy trials occurred in patient populations unselected for their specific genomic alterations which, in turn, may have significantly diluted the targeted agent’s clinical efficacy^11^.

Several studies suggest that matching genomic alterations to targeted therapy can improve outcomes^12–15^. However, it is plausible that, in malignancies such as pancreatic cancer, there may be more than one genomic driver. One strategy to overcome this challenge is to treat with combinations of matched agents, with the intention to target multiple genomic alterations at once^16^.

Here, we describe a cohort of patients with advanced pancreatic adenocarcinoma who were treated with individualized matched therapy as part of our precision medicine program after discussion at our molecular tumor board. An illustrative case given a chemotherapy-free regimen of targeted agents highly matched to her genomic alterations achieved a partial remission lasting 17.5 months. Our observations suggest that larger cohorts of patients with advanced pancreatic cancer should be studied prospectively with a precision paradigm approach.

## Results

### Baseline characteristics

**Fig. 1:**
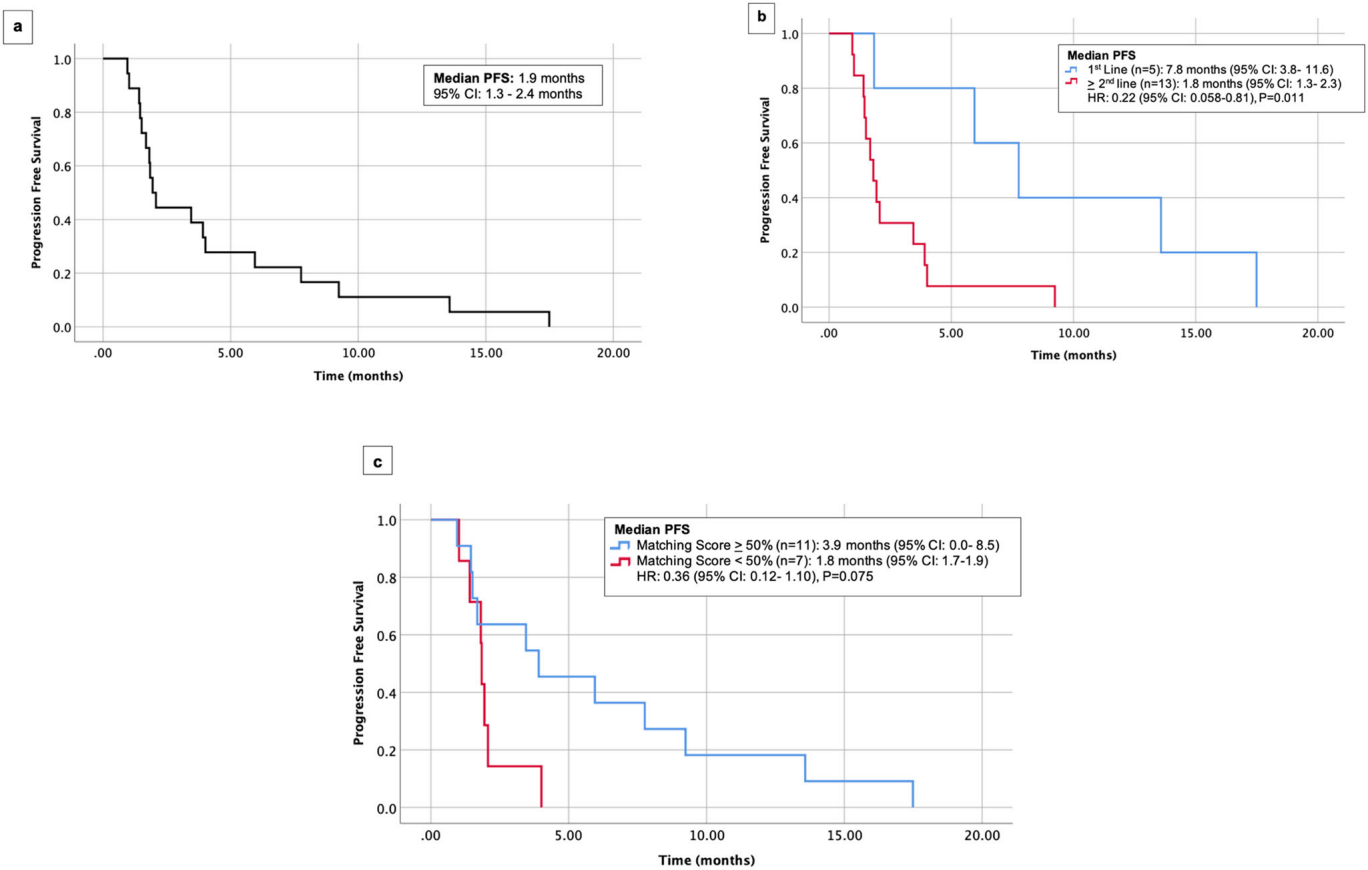
Progression-free survival (PFS) among 18 patients with pancreatic cancer who received matched therapy. **a** PFS in 18 patients. **b** PFS in 5 patients who received targeted therapy as first line versus 13 patients who received it as ≥2nd line. **c** PFS in 11 patients with matching score ≥50% versus 7 patients with matching score <50%. CI confidence interval, PFS progression-free survival.

**Fig. 2:**
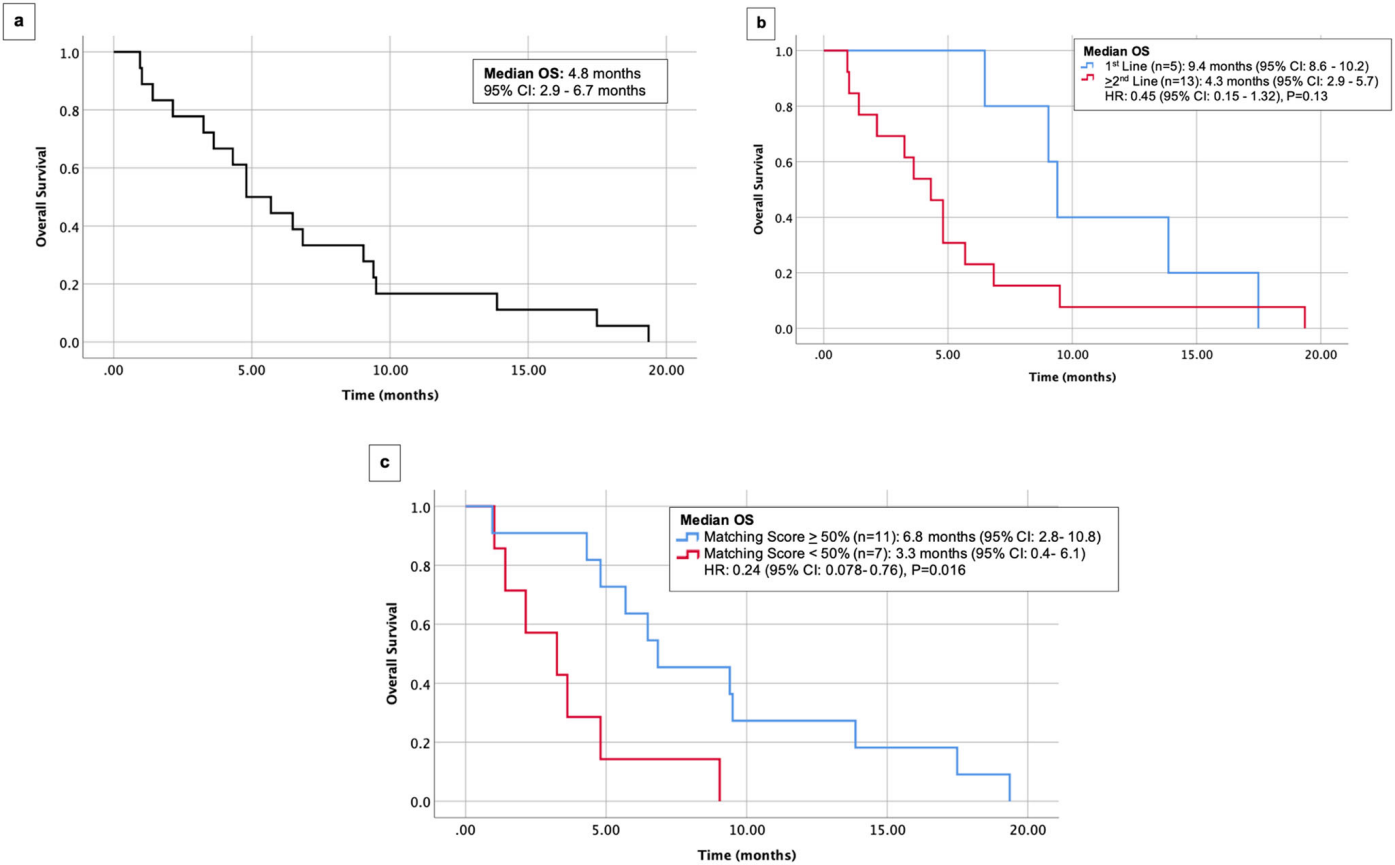
Overall survival (OS) among 18 patients with pancreatic cancer who received matched therapy. **a** OS in 18 patients. **b** OS in 5 patients who received targeted therapy as first line versus 13 patients who received it as ≥2nd line. **c** OS in 11 patients with matching score ≥50% versus 7 patients with matching score <50%. CI confidence interval, OS overall survival.

**Fig. 3:**
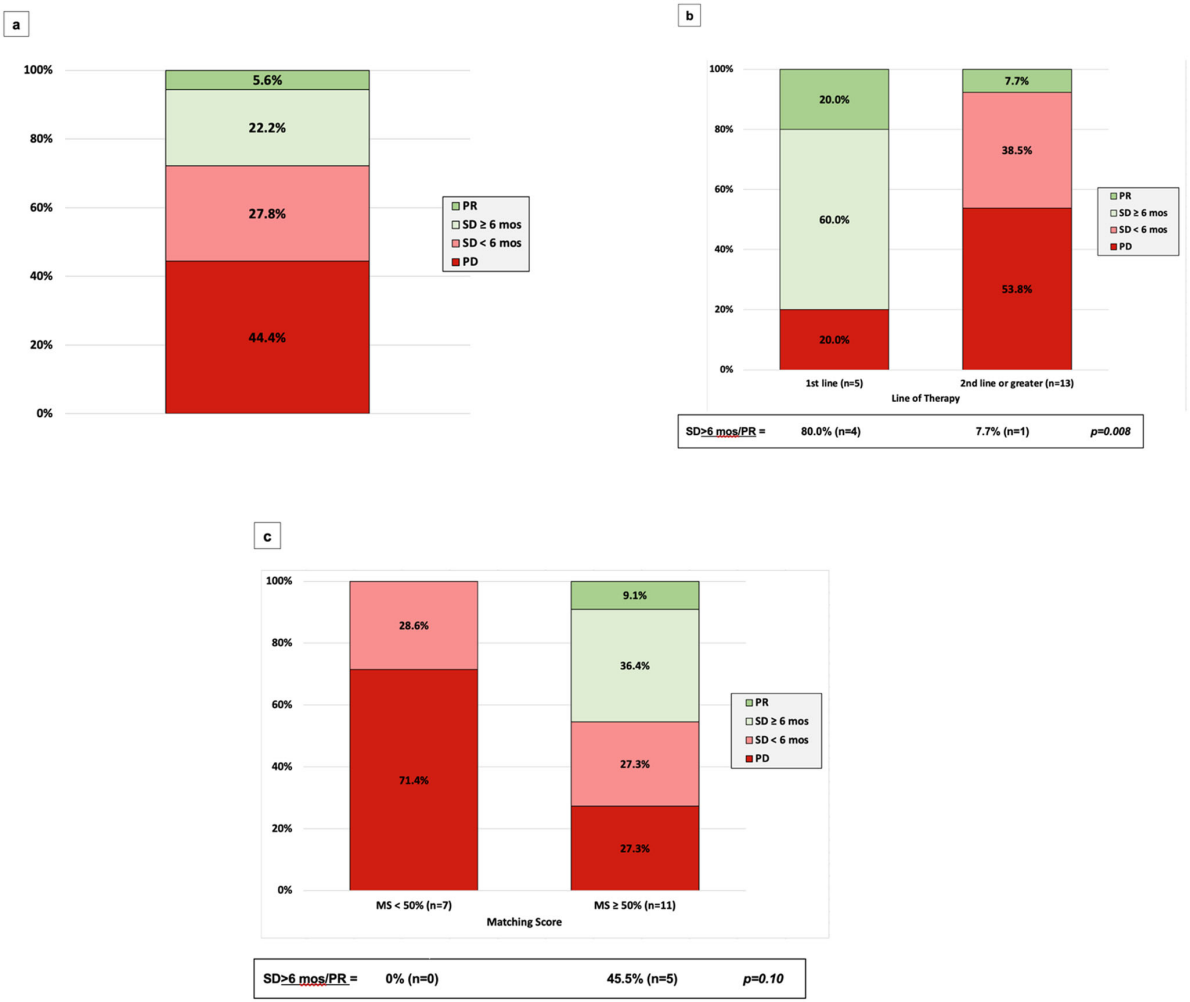
Clinical benefit and objective response rate among 18 patients with pancreatic cancer who received matched therapy. **a** Clinical benefit (SD ≥ 6 months/PR) and objective response rate in 18 patients. **b** Clinical benefit (SD ≥ 6 months/PR) and objective response rate in 5 patients who received targeted therapy as first line versus 13 patients who received it as ≥ 2nd line. **c** Clinical benefit (SD ≥ 6 months/PR) and objective response rate in 11 patients with matching score ≥50% versus 7 patients with matching score <50%. MS matching score, PR partial response, SD stable disease.

Of 6831 patients in the UCSD institutional PREDICT database, 18 patients with advanced pancreatic cancer who received at least one matched targeted therapy (immunotherapy excluded) were identified (Supplementary Fig. 1). At the time of matched therapy initiation, 88.9% (*N* = 16/18) had metastatic disease (while the others had advanced unresectable disease) and 72.2% (*N* = 13/18) of the cohort had received prior lines of therapy, predominately cytotoxic chemotherapy (Table 1). The median number of therapies prior to treatment with matched therapy was one. With regard to the genomic-sequencing data, 83.3% of patients (*N* = 15/18) underwent tissue NGS and, while several platforms were utilized, the majority of samples were tested with the FoundationOne CDx assay. Blood-based ctDNA NGS testing was performed in 77.8% of patients (*N* = 14/18), with the majority of samples run by Guardant360 ctDNA assay. Outcomes are shown in Figs. 1–3.

**Table 1:** Patient demographics and genomic characteristics.

Baseline characteristic (*N* = 18)	*N* (%)
Age at initiation of matched therapy (median), years	67 (47–84)
Sex	
Male, *N* (%)	7 (38.9%)
Female, *N* (%)	11 (61.1%)
Race	
White, *N* (%)	15 (83.3%)
Hispanic, *N* (%)	1 (5.6%)
Asian, *N* (%)	1 (5.6%)
Black, *N* (%)	1 (5.6%)
Extent of disease at diagnosis	
Localized, *N* (%)	6 (33.3%)
Locally advanced, *N* (%)	2 (11.1%)
Metastatic, *N* (%)	10 (55.6%)
Extent of disease at time of matched therapy	
Locally advanced, *N* (%)	2 (11.1%)
Metastatic, *N* (%)	16 (88.9%)
Number of prior therapies, median (range)	1.5 (0–4)
Matched therapy given as first-line therapy, *N* (%)	5 (27.8%)
Matched therapy given as second line or greater, *N* (%)	13 (72.2%)
Number of matched targeted agents, median (range)	2.5 (1–4)
Therapies given prior to matched treatment (*N* = 13), *N* (%)	
Gemcitabine/nab-paclitaxel	9 (50%)
FOFLIRINOX	6 (33.3%)
Clinical Trial	3 (16.7%)
Capecitabine	2 (11.1%)
FOLFOX	1 (5.6%)
5-FU/liposomal irinotecan	2 (11.1%)
Gemcitabine/erlotinib	1 (5.6%)
Gemcitabine	1 (5.6%)
Genomic profiling	
Tissue NGS obtained, *N* (%)	15 (83.3%)
Blood ctDNA obtained, *N* (%)	14 (77.8%)
Both ctDNA and tissue NGS obtained, *N* (%)	11 (61.1%)
Tissue NGS Platforms^a^ (*N* = 15)	
Foundation One^b^	14 (77.8%)
Tempus^b^	1 (5.6%)
Institutional Assay (UCSD)	1 (5.6%)
ctDNA Platforms (*N* = 14)	
Guardant360	13 (72.2%)
Tempus	1 (5.6%)
Matching score, median (range)	50% (14–100%)

*ctDNA* circulating-tumor DNA, *FOLFIRINOX* 5-fluorouracil, oxaliplatin, irinotecan, leucovorin, *FOLFOX* 5-fluorouracil, oxaliplatin, leucovorin, *NGS* next-generation sequencing.

^a^Testing laboratories:

Caris Life Sciences, https://www.carismolecularintelligence.com/molecular-testing-services/; Foundation One and Foundation ACT, https://www.foundationmedicine.com/; Guardant360, http://www.guardant360.com/; Tempus, https://www.tempus.com/genomic-sequencing/.

^b^One patient underwent both Foundation One and Tempus NGS.

### Patients with higher degrees of genomic matching had longer OS

There was no significant difference in age, gender, or number of patients receiving first line versus later lines of therapy in patients with matching scores <50 versus ≥50% (Supplementary Table 1).

Median OS of the cohort was 4.8 months (Fig. 2a). When stratified by line of therapy, median OS was 9.4 months versus 4.3 months (*P* = 0.13) for patients treated in the first line versus second line or later, respectively (Fig. 2b). (Patients treated with chemotherapy also do better in first versus second line of therapy). Median OS was 6.8 and 3.3 months (*P* = 0.016*)* for a matching score of ≥50 versus <50%, respectively (Fig. 2c).

### Patients with higher degrees of genomic matching trended towards longer PFS and higher CBR

Among the cohort of 18 patients, median PFS was 1.9 months (Fig. 1a). When stratified by matching score, dichotomized by a score of ≥50 versus <50%, median PFS was 3.9 versus 1.8 months (*P* = 0.075) (Fig. 1c). When stratified by line of therapy in terms of treatment with matched therapy in the first line versus the second line or later, median PFS was 7.8 months versus 1.8 months (*P* = 0.011), respectively (Fig. 1b).

The clinical benefit rate ([CBR], SD ≥ 6 months/PR/CR) was 27.8% (5 of 18 patients). All five of these patients received regimens that were chemotherapy-free and all five had a matching score ≥50%. CBR for patients with a matching score of <50% was 0 and 45.5% in patients with a matching score ≥50% (*N* = 5/11) (*P* = 0.10) (Fig. 3c). When stratified by line of therapy, the CBR for patients treated in the first line was 80.0% (four of five patients) and 7.7% (1 of 13 patients) for patients treated in the second line or later (*P* = 0.008) (Fig. 3b).

### Toxicity

Among the cohort, no grade 3 or 4 drug-related toxicities were noted with the matched therapy regimens when dosed according to Methods. The most common grade 1 to 2 toxicities were rash (seen with trametinib) and diarrhea (seen with erlotinib and trastuzumab and cetuximab), diarrhea (seen with trametinib) and myelosuppression (seen with palbociclib). At the doses used (see Methods), only the trametinib and everolimus combination required early discontinuation for chronic side effects (rash/mucositis).

### Illustrative case among patients achieving partial response to matched therapy

Table 2^17–19^ lists patients who achieved clinical benefit (CBR, defined as SD ≥ 6 months/PR/CR).

**Table 2:** Clinical and genomic characteristics of patients who achieved either stable disease ≥6 months or partial response (*N* = 5 patients).

Patient ID# Age (in years)/sex	Matched drugs and relevant pathogenic genomic alterations	Matching Score (%) ≥50% versus <50%	PFS (approximate months)	OS (months)	Best response	Comments
#20 (69 y/M)	Palbociclib, *CDKN2A* loss exons 1–2, *CDKN2B* loss, Trametinib, *KRAS* G12D, *KRAS* G12R, Bevacizumab, *TP53* R267W	≥50%	6.0	6.5	SD ≥ 6 months	
#12 (74 y/F)	Trametinib, *BRAF* V600E, *SMAD4* loss, Trastuzumab and lapatinib, *ERBB2* amplification, Bevacizumab, *TP53* V218E	≥50%	7.8	9.4	SD ≥ 6 months	
#24 (63 y/F)	Palbociclib, *CDKN2A*_p16 R80* Trametinib, *KRAS* G12D	≥50%	9.2	19.4	SD ≥ 6 months	Afatinib also given because *KRAS* alterations may require EGFR pathway in pancreatic cancer^17^
#22 (82 y/F)	Trametinib, *GNAS* R201C, *KRAS* G12D, *NF1* D1976fs	≥50%	13.6	13.9	SD ≥ 6 months	*GNAS, KRAS* and *NF1* alterations all activate the MEK pathway^18,19^; trametinib is a MEK inhibitor
#18 (63 y/F)	Palbociclib, *CDKN2A* loss exons 1–2, *CDKN2B* loss Trametinib, *KRAS* G12D, *KRAS* G12R, *SMAD4* deletion exon 11 Bevacizumab, *TP53* R267W.	≥50%	17.5+	17.5	PR	

See Supplementary Table 2 for 13 patients who did not achieve clinical benefit.

*cfDNA* cell free DNA, *F* female, *M* male, *NGS* next-generation sequencing, *PFS* progression-free survival, *TMB* tumor mutational burden (mutations/megabase), *MSS* microsatellite stable, *y* years.

**Fig. 4:**
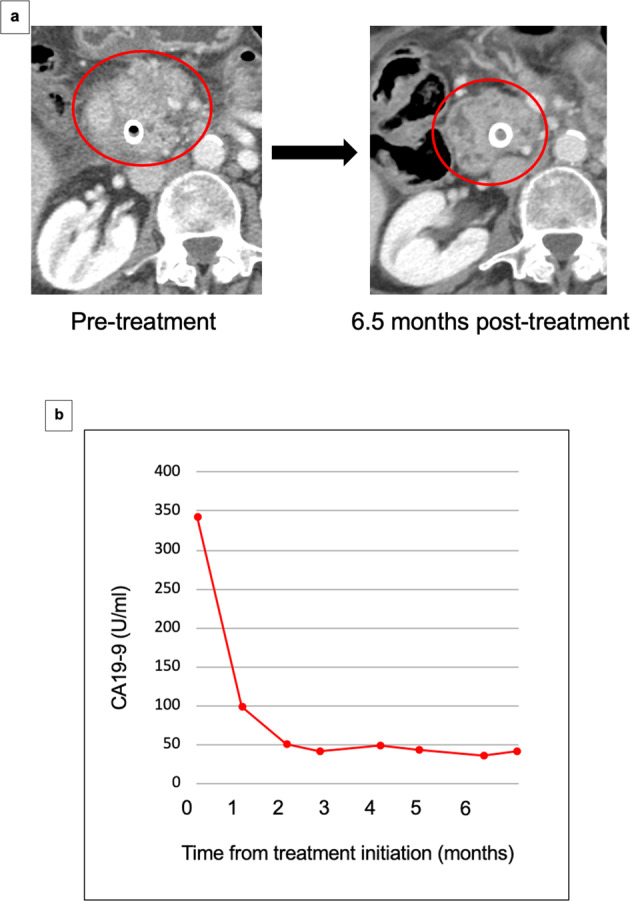
Illustrative case #18 (see Table 2). Sixty-four-year-old woman with pancreatic cancer with *KRAS G12D, KRAS G12R, CDKN2A loss exons 1–2, CDKN2B loss, SMAD4* deletion exon 11, *TP53 R267W* on tissue NGS treated with palbociclib (targets CDK4/6 upregulated by *CDKN2A/B* loss), trametinib (targets MEK, upregulated by *KRAS* and *SMAD4* mutations), and bevacizumab (targets VEGF, upregulated by *TP53* mutations). There were no serious drug-related side effects. She achieved partial response with PFS of 17.5 months. Patient died from complications of a chronic obstructive pulmonary disease exacerbation, which was felt to be unrelated to her cancer or her therapy. At the time of death, patient was free from progression. **a** Serial CT scans of primary pancreatic mass. **b** CA-19-9 trend on therapy (reference range, 30–42 U/mL).

The longest responder was patient #18, a 65-year-old woman with a history of chronic obstructive pulmonary disease and hypertension who was diagnosed with de novo metastatic pancreatic adenocarcinoma with lung metastases (Fig. 4). Her tissue NGS showed *KRAS* G12D*, KRAS* G12R*, CDKN2A* loss exons 1–2, *CDKN2B* loss, *SMAD4* deletion exon 11, *TP53* R267W. Based on the patient’s genomic profiling, the patient was started on matched targeted therapy with the MEK inhibitor trametinib (1 mg orally daily) for *SMAD4* and *KRAS* alterations both of which activate the MEK pathway^20–22^, the CDK4/6 inhibitor palbociclib (75 mg orally 3 weeks on, 1 week off) for *CDKN2A* exons 1–2 loss and *CDKN2B* loss which can upregulate CDK4/6^23^, and the VEGF-A antibody bevacizumab (7.5 mg/kg intravenously every 3 weeks) for *TP53*, which can activate the VEGF/VEGFR pathway^24,25^. On matched therapy, her CA-19-9 decreased significantly (Fig. 4b) from a peak level of 349 to a nadir level of 35 (normal range, 30–42 U/mL) and scans showed ~37% regression of pancreatic and lung metastases (Fig. 4a). Her partial response lasted 17.5 months without progression of disease. However, the patient passed away due to the complications of underlying chronic lung disease. There were no serious drug-related side effects.

## Discussion

There have been limited successes with the use of targeted therapy in pancreatic cancer, perhaps because only a minority of pancreatic cancer trials use a biomarker for enrolling patients^26^. It is clear that a biomarker-driven approach has driven advances for other cancers. Given clinical responses seen with N-of-One combination matched therapy in the tumor-agnostic I-PREDICT trial^16^, there was an interest in this approach in a pancreatic cancer cohort.

The population of patients evaluated in this study was diverse, and the majority of individuals had received prior lines of systemic therapy. With matched targeted therapy, there was significantly longer overall survival among patients with a high matching score, reflecting a high degree of matching, compared to those with a low degree of matching of drugs to molecular alterations. A similar trend was observed with improved PFS and CBR (SD ≥ 6 months/PR/CR). When stratified by line of matched therapy, PFS and CBR were significantly better among patients treated with matched therapy as first-line therapy compared to those treated in the second line and beyond. Similar trends were seen with overall survival, although the overall survival differences in first versus second line or greater did not reach statistical significance. There were no grade 3–4 toxicities at least possibly drug related reported with the matched therapy combinations administered. The rates of OS and PFS reported among this cohort, particularly in the first line and high matching score cohorts, are notable as they are similar to those seen with cytotoxic chemotherapy: FOLFIRINOX with median OS of 11.1 months, and nab-paclitaxel plus gemcitabine with median OS of 8.5 months^1,2,27^. However, without a randomized trial, these results are not comparable.

Altogether, five of 18 patients achieved clinical benefit (SD ≥ 6 months/PR/CR). These patients were treated with chemotherapy-free regimens. The key similarity among these five patients with diverse genomic alterations and therapies was a higher degree of matching of genomic alterations to targeted therapy. From this study and the larger I-PREDICT trial^16^, the ability to match therapy to a high proportion of detected alterations appears to be a significant factor in the efficacy of matched therapy. Of note, patient #22 (PFS = 13.6 months) was treated with trametinib monotherapy, an agent which failed to show benefit in combination with gemcitabine in a biomarker unselected population^6^; the molecular alterations in this patient included anomalies in *GNAS, KRAS*, and *NF1*, all of which can activate the MEK pathway^28–31^. Hence, this patient had multiple activating mutations in the MEK/ERK pathway, perhaps explaining their response to trametinib^32^. This is supported by reports of benefit with trametinib in gastrointestinal malignancies harboring GNAS alterations^19^.

More recently, there have been attempts at biomarker-driven clinical trials in advanced pancreatic cancer. For example, the phase III POLO trial tested the PARP inhibitor olaparib versus placebo as maintenance therapy in patients with germline BRCA1/2 alterations who achieved at least stable disease after cytotoxic chemotherapy induction. The trial showed progression-free survival (PFS) benefit with the addition of olaparib maintenance^10^. While the results of this trial are promising and practice changing, the utility of olaparib is limited to a particular subset of *BRCA*-mutant patients who have a favorable response to cytotoxic chemotherapy^10^.

Another example is the phase I pan-cancer trial of *KRAS* G12C inhibitor sotorasib enrolled ten pancreatic cancer patients whose tumors harbored *KRAS G12C* alterations. Stable disease was seen in six patients (60%), while four (40%) had progressive disease^33^. The TAPUR trial treated patients with pancreatic cancer harboring *CDKN2A* loss or mutation with the CDK4/6 inhibitor palbociclib, but failed to show any clinical response with palbociclib monotherapy^34^. As mentioned above, PARP inhibitors in germline *BRCA1/2-*mutated pancreatic cancer has been one targeted therapy success. The rate of germline DNA damage repair alterations in *ATM, BRCA1*, and *BRCA2* has been noted to be as high as 10% in patients with advanced pancreatic cancer^35^ and these alterations confer sensitivity to PARP inhibitors. Lastly, *NRG1* gene fusions in *KRAS* wild-type pancreatic adenocarcinoma may be a clinically meaningful target^36^, as neuroregulin family proteins such as NRG1 act on the EGFR receptors. Although *NRG1* fusions are rare, there are reports of clinical responses to afatinib HER2/HER3 inhibition in pancreatic cancer^36,37^.

A previous study implemented a precision oncology approach for patients with pancreatic cancer giving targeted therapies based on patients NGS reports^38^. This approach reported a significant PFS benefit in patients who received a targeted therapy in addition to chemotherapy; however, there were no combination therapies given. The authors of this study reported a median of four alterations per NGS report. The previously reported study proved that it was feasible to perform NGS on pancreatic tumors and to give patients with pancreatic cancer targeted therapies and improve PFS; our study builds on such experience by also assessing matching scores and combination targeted therapies.

There are several key limitations of our study. This was a pilot study with a small group of patients; therefore, these results require prospective validation with a larger randomized cohort. Moreover, given that therapy selection is based on a patient’s unique genomic profile, the benefit of targeting a specific set of alterations inherently differs from patient to patient. Even so, the strategy of combinatorial matched therapy among a group of patients with differing combinations of alterations has been shown to be a viable strategy across many tumor types, enhanced by molecular tumor board discussions^13,39–41^. While this study excluded patients treated with checkpoint inhibitors, a recent study showed that, among pancreatic cancer patients with alterations in chromatin remodeling genes, treatment with immunotherapy was associated with response^42^. Given these data, immunotherapy may also have a role in matched therapy in pancreatic cancer. Subsequent lines of therapy may also influence survival, and ultimately a randomized trial is warranted. Finally, more research is needed, as many patients did not respond or responded inadequately, especially in later lines of therapy. Methodologies such as transcriptomics, immunomics, and proteomics should be explored, in order to uncover additional molecular drivers and better matched therapeutic options and to better understand resistance mechanisms in pancreatic cancer, especially in patients whose tumors are refractory to prior treatment regimens.

Matched targeted therapy may offer a more tolerable toxicity profile compared to cytotoxic chemotherapy and may be a better suited option for patients with marginal performance status or organ dysfunction who would otherwise be poor chemotherapy candidates. The results of this analysis suggest that, when genomic-directed matched therapy can achieve a high degree of matching, and especially in first-line settings, clinical outcomes can be improved, even with regimens that exclude chemotherapy. These observations support our prior reports that combinations of targeted agents, such as matched CDK4/6 inhibitors and MEK inhibitors (given when cognate pathway co-alterations such as *CDKN2A/B* loss and *KRAS* mutations are present), may have activity, even when single agents are ineffective^43^. The current results also reflect the need for implementation of multi-omic and functional testing for all patients with advanced pancreatic cancer, perhaps earlier in the course of the disease, to further identify actionable alterations^26,44^. Prospective trials of this strategy are warranted.

## Methods

### Patients

This was a single-center analysis of real-world patients with advanced pancreatic cancer treated with matched therapy at the University of California San Diego (UCSD) Moores Cancer Center for Personalized Cancer Therapy. The patients were analyzed according to the guidelines of the PREDICT (Profile Related Evidence Determining Individualized Cancer Therapy) protocol (NCT02478931) and any investigational interventions/therapies for which all patients gave written informed consent. Protocols were approvaed by the UCSD Internal Review Board. Patients underwent genomic profiling of tissue (somatic) and/or blood using next-generation sequencing (NGS) and were treated with targeted therapy based on their individual genomic profiling. The turnaround time for an NGS report was roughly 3–4 weeks. All patients’ genomic profiling were reviewed at a Molecular Tumor Board (MTB) where the targeted therapy regimen was suggested based on the basis of the MTB expert opinion as well as published guidelines such as OncoKB (https://www.oncokb.org/)^22,39^. The UCSD MTB is a tumor-agnostic (electronic and face-to-face) tumor board comprised of medical oncologists, radiation oncologists, surgeons, radiologists, pathologists, basic scientists, bioinformatics specialists, clinical study coordinators, patient navigators, and drug acquisition specialists^39^ that focuses on discussing therapies based on patients’ tumor multi-omic results^45^. However, final treatment choices were the prerogative of the physician who was managing the patient. As previously described in the I-PREDICT trial^16^, patients were started at ~50% of the usual dose for two-drug combinations and at ~33% of the usual dose for three-drug combinations to avoid overlapping toxicities^46^. Doses were escalated to tolerance by the individual oncologist. Evaluable patients had at least one follow up visit. Patients treated with immunotherapy were excluded from this analysis (Supplementary Fig. 1). Patients treated had ECOG Performance Status Scale 0–2. Targeted therapies were obtained via the MTB drug acquisition specialists through insurance approval (i.e., prior authorization approval, denial appeal approvals), patient assistance subsidy programs through the manufacturer, or as compassionate use donated from the manufacturers. Patients could also be navigated to secondary clinical trials. Individual patient toxicities were assessed on approximately a weekly basis utilizing the Common Terminology Criteria for Adverse Events (CTCAE) v3.0 toxicity scoring system.

### Next-generation sequencing (NGS)

NGS was completed by commercially available clinical laboratory improvement amendment (CLIA) platforms including Foundation One (343–352 genes) (https://www.foundationmedicine.com), Caris (140 genes) (https://www.carislifesciences.com), Tempus (595 genes) (https://www.tempus.com), and a University of California San Diego institutional assay (397 genes). Although specific gene alterations analyzed differ between each assay, there is a strong degree of overlap^47,48^.

Blood derived circulating-tumor DNA (ctDNA) NGS analyses were done through Guardant Health (73 genes) (https://guardant360.com) and Foundation One Liquid (67–77 genes) (https://www.foundationmedicine.com)^32,49–51^. Only non-synonymous alterations that were not variants of unknown significance were analyzed in this study.

### Endpoints, statistical methods, matching score, and case studies

Descriptive statistics were used to summarize the patient characteristics. Key endpoints of the study included OS, PFS, objective response rate (ORR), and CBR (defined as stable disease (SD) ≥ 6 months or partial response (PR) or complete response (CR)). OS was calculated from the time of initiation of therapy to death or last follow up. PFS was calculated from the time of initiation of targeted therapy to progression or death. First therapy after MTB was considered. OS and PFS were stratified by line of matched therapy (1st line vs 2nd line or greater) and matching score (<50 vs ≥50%). Survival analysis was done using Kaplan–Meier analysis and stratified survival curves were compared using the log-rank test. Patients still progression-free or alive at last follow up for PFS and OS, respectively, were censored on that date. ORR and progression of disease were defined by RECIST v1.1 per physician assessment^52^. The CBR was compared between subgroups using Fishers exact test. As previously described in detail^11,16^, the matching score roughly describes the proportion of targeted alterations over the total number of deleterious alterations detected; it reflects the degree to which drugs are matched to genomic alterations. Matching score was determined by investigators who were blinded to outcome at the time of calculation. Kaplan–Meier analysis and log-rank test were used to compare subgroups of patients. *P*-values ≤ 0.05 were considered significant. Statistical analyses were performed with SPSS version 25 software (IBM Corporation, Armonk, NY).

### Reporting summary

Further information on research design is available in the Nature Research Reporting Summary linked to this article.

## Supplementary information

SUPPLEMENTAL MATERIAL

Reporting Summary

## Data Availability

The data that support the findings of this study are available in Table 2 and Supplementary Table 2. For further information please contact the corresponding authors. Next-generation sequencing was performed by the following CLIA-certified labs: Foundation Medicine, Caris Life Sciences, Tempus, and Guardant 360. Accessing sequencing data beyond what was published would require a data usage agreement with the corresponding commercial company.

## Code availability

Statistical analyses were performed with SPSS version 25 software (IBM Corporation, Armonk, NY).
