# Supplementary material for: Personalized matched targeted therapy in advanced pancreatic cancer: a pilot cohort analysis
Source: NPJ Genom Med. 2023 Jan 20;8:1. doi: 10.1038/s41525-022-00346-5 (PMC9860045; doi:10.1038/s41525-022-00346-5)

Supplementary Table 1: Comparison of characteristics for patients with matching score <50% versus ≥50% and first versus second line or greater.

**Characteristics**	**Matching Score <50% (N = 7)**	**Matching Score ≥50% (N = 11)**	**P value**
**Age > 67 years* (N (%))**	3 (42.8%)	6 (54.5%)	1.0
**Number of men (N (%))**	3 (42.9%)	4 (36.4%)	1.0
**Matched therapy as first line therapy (N (%))**	1 (14.2%)	4 (36.4%)	0.60
	**1st line of therapy**	**Later lines**	
**Matching score ≥ 50%**	4/5 (80%)	7/13 (54%)	0.3

*Age 67 was the median age of the overall cohort.

Supplementary Table 2: Molecular alterations and agents administered for 13 patients who did not achieve clinical benefit

**Molecular alterations**	**Drugs Administered**	**Matching Score (%) ≥50% versus <50%**
CDKN2A alteration<br>*KRASG12V*<br>*TP53* mutation	Palbociclib and trametinib	≥50%
CDKN2A alteration<br>GNASR201H<br>KRAS G12D<br>TP53 mutation	Palbociclib and trametinib	≥50%
CDKN2A alteration<br>*ERBB2* amplification<br>*KRAS G12D*	Palbociclib and trametinib	≥50%
CDKN2A alteration<br>*GNASR201C*<br>*KRAS Q61H*<br>*SMAD4* alteration	Palbociclib and trametinib	≥50%
Alterations in<br>*CDKNA, CCND1, CCNE1, KRAS*	Palbociclib, trametinib, and bevacizumab	≥50%
Alterations in *CDKN2A, KRAS, TP53*	Palbociclib, trametinib, and bevacizumab	≥50%
MDM2 amplification<br>Alterations in *AKT2, AXL, KRAS, TP53,*	Trametinib and everolimus	<50%
CDKNA loss<br>Alterations in *BRCA2,*<br>*IRS2, KRAS. SMAD4, TP53*	Olaparib and trametinib	<50%
Alterations in<br>*BRCA1, KRAS, MEN1, MSH6*	Oxaliplatin* and capecitabine	<50%
CDKNA loss<br>Alterations in *ARID1A, BRCA1, CDKN1B, ERBB2, KRAS, TP53*	Olaparib	<50%
Alteration in *ATM, CCND2, CDH1, FGFR2, FGF23, GNAS, MYC, NOTCH1, SMAD4, TP53*	Erlotinib, cetuximab, palbociclib, and trametinib	<50%
Alterations in<br>*CDK4, CDKN2A, ERBB2, ERBB3, GLi1, KRAS, TP53*	Trastuzumab and pertuzumab	<50%
BRAF V600E<br>Alterations in *CDH1, TP53,*	Trametinib and erlotinib	<50%

*Platinum agents target DNA repair and hence were considered as matched targeted agents for *BRCA*-mutant tumors

Supplementary Figure 1: Consort Diagram: A total of 123 patients with pancreatic cancer had NGS results in the database.

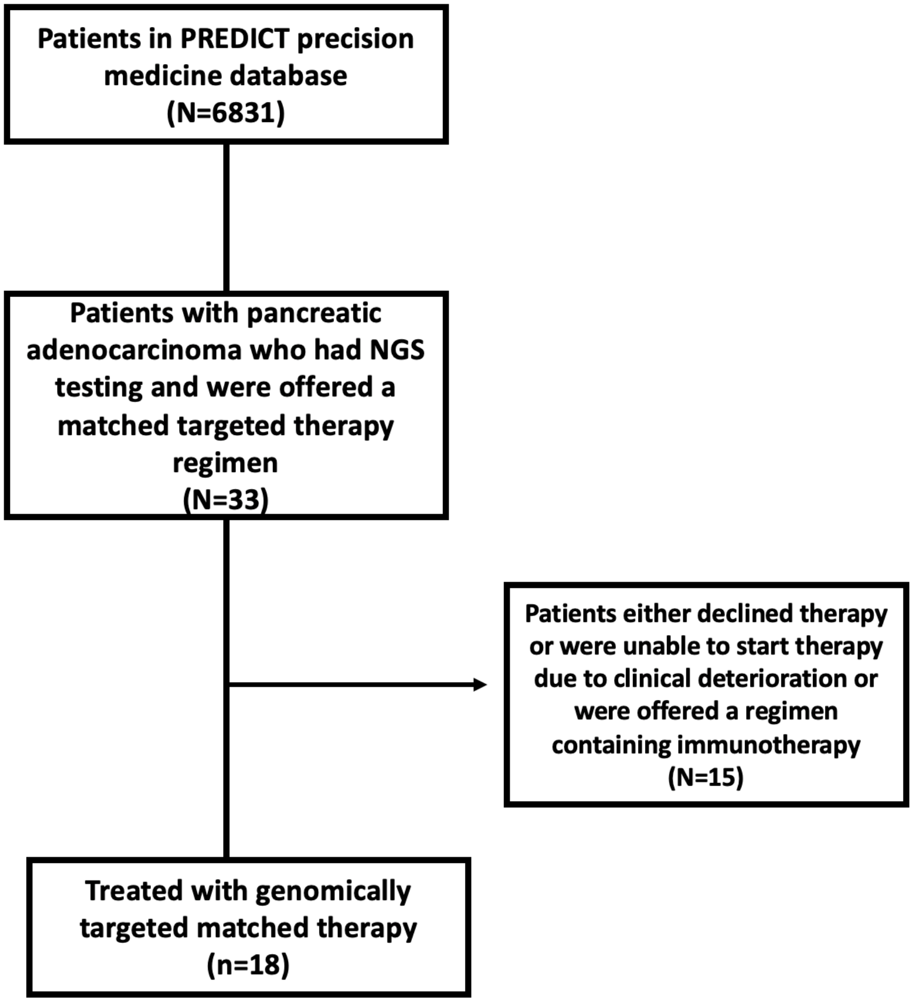

Supplement: Supplementary file 1 — SUPPLEMENTAL MATERIAL [file 41525_2022_346_MOESM1_ESM.pdf]
