# Supplementary material for: Personalized matched targeted therapy in advanced pancreatic cancer: a pilot cohort analysis
Source: NPJ Genom Med. 2023 Jan 20;8:1. doi: 10.1038/s41525-022-00346-5 (PMC9860045; doi:10.1038/s41525-022-00346-5)
Supplement: Supplementary file 2 — Reporting Summary [file 41525_2022_346_MOESM2_ESM.pdf]

nature portfolio

Corresponding author(s): NPJGENMED-01670R

Last updated by author(s): Oct 25, 2022

Reporting Summary

Nature Portfolio wishes to improve the reproducibility of the work that we publish. This form provides structure for consistency and transparency in reporting. For further information on Nature Portfolio policies, see our Editorial Policies and the Editorial Policy Checklist.

Statistics

For all statistical analyses, confirm that the following items are present in the figure legend, table legend, main text, or Methods section.

n/a	Confirmed	
☐	☒	The exact sample size (*n*) for each experimental group/condition, given as a discrete number and unit of measurement
☐	☒	A statement on whether measurements were taken from distinct samples or whether the same sample was measured repeatedly
☐	☒	The statistical test(s) used AND whether they are one- or two-sided<br>*Only common tests should be described solely by name; describe more complex techniques in the Methods section.*
☐	☒	A description of all covariates tested
☐	☒	A description of any assumptions or corrections, such as tests of normality and adjustment for multiple comparisons
☐	☒	A full description of the statistical parameters including central tendency (e.g. means) or other basic estimates (e.g. regression coefficient) AND variation (e.g. standard deviation) or associated estimates of uncertainty (e.g. confidence intervals)
☒	☐	For null hypothesis testing, the test statistic (e.g. *F*, *t*, *r*) with confidence intervals, effect sizes, degrees of freedom and *P* value noted<br>*Give P values as exact values whenever suitable.*
☐	☒	For Bayesian analysis, information on the choice of priors and Markov chain Monte Carlo settings
☐	☒	For hierarchical and complex designs, identification of the appropriate level for tests and full reporting of outcomes
☒	☐	Estimates of effect sizes (e.g. Cohen's *d*, Pearson's *r*), indicating how they were calculated

Our web collection on statistics for biologists contains articles on many of the points above.

Software and code

Policy information about availability of computer code

Data collection	No software was used
Data analysis	Statistical analyses were performed with SPSS version 25 software (IBM Corporation, Armonk, NY).

For manuscripts utilizing custom algorithms or software that are central to the research but not yet described in published literature, software must be made available to editors and reviewers. We strongly encourage code deposition in a community repository (e.g. GitHub). See the Nature Portfolio guidelines for submitting code & software for further information.

Data

Policy information about availability of data

All manuscripts must include a data availability statement. This statement should provide the following information, where applicable:

- Accession codes, unique identifiers, or web links for publicly available datasets
- A description of any restrictions on data availability
- For clinical datasets or third party data, please ensure that the statement adheres to our policy

The data that support the findings of this study are available from the corresponding author upon reasonable request.

Human research participants

Policy information about studies involving human research participants and Sex and Gender in Research.

Reporting on sex and gender	Descriptive statistics were used to summarize the patient characteristics.
Population characteristics	Patients underwent genomic profiling of tissue (somatic) and/or blood using next generation sequencing (NGS) and were treated with targeted therapy based on their individual genomic profiling.
Recruitment	This was a single-center analysis of real-world patients with advanced pancreatic cancer treated with matched therapy at the University of California San Diego (UCSD) Moores Cancer Center for Personalized Cancer Therapy.
Ethics oversight	all ethical approvals from the UCSD Internal Review Board.

Note that full information on the approval of the study protocol must also be provided in the manuscript.

Field-specific reporting

Please select the one below that is the best fit for your research. If you are not sure, read the appropriate sections before making your selection.

☒ Life sciences ☐ Behavioural & social sciences ☐ Ecological, evolutionary & environmental sciences

For a reference copy of the document with all sections, see nature.com/documents/nr-reporting-summary-flat.pdf

Life sciences study design

All studies must disclose on these points even when the disclosure is negative.

Sample size	No sample-size calculation was performed.
Data exclusions	Patients still progression free or alive at last follow up for PFS and OS, respectively, were censored on that date.
Replication	No reproducibility as this were all n of 1 therapies based on matching scores.
Randomization	No randomization as all patients were treated by the molecular tumor board.
Blinding	Matching score was determined by investigators who were blinded to outcome at the time of calculation.

Reporting for specific materials, systems and methods

We require information from authors about some types of materials, experimental systems and methods used in many studies. Here, indicate whether each material, system or method listed is relevant to your study. If you are not sure if a list item applies to your research, read the appropriate section before selecting a response.

Materials & experimental systems

n/a	Involved in the study	
☒	☐	Antibodies
☒	☐	Eukaryotic cell lines
☒	☐	Palaeontology and archaeology
☒	☐	Animals and other organisms
☐	☒	Clinical data
☒	☐	Dual use research of concern

Methods

n/a	Involved in the study	
☒	☐	ChIP-seq
☒	☐	Flow cytometry
☒	☐	MRI-based neuroimaging

Clinical data

Policy information about clinical studies

All manuscripts should comply with the ICMJE guidelines for publication of clinical research and a completed CONSORT checklist must be included with all submissions.

Clinical trial registration	NCT02478931
Study protocol	https://clinicaltrials.gov/ct2/show/NCT02478931

Data collection

This was a single-center analysis of real-world patients with advanced pancreatic cancer treated with matched therapy at the University of California San Diego (UCSD) Moores Cancer Center for Personalized Cancer Therapy. The

Outcomes

Key endpoints of the study included OS, PFS, objective response rate (ORR), and CBR (defined as stable disease (SD) > 6 months or partial response (PR) or complete response (CR)). OS was calculated from the time of initiation of therapy to death or last follow up. PFS was calculated from the time of initiation of targeted therapy to progression or death. First therapy after MTB was considered. OS and PFS were stratified by line of matched therapy (1st line vs 2nd line or greater) and matching score (< 50% vs > 50%). Survival analysis was done using Kaplan-Meier analysis and stratified survival curves were compared using the log-rank test.
